# The Missing Link: Why Value-Based Healthcare Needs Healthcare and Management Science to Unite Efforts: Comment on "Reflections on Managing the Performance of Value-Based Healthcare: A Scoping Review"

**DOI:** 10.34172/ijhpm.9121

**Published:** 2025-07-12

**Authors:** Dorine van Staalduinen, Paul van der Nat

**Affiliations:** ^1^Martini Hospital, Groningen, The Netherlands.; ^2^Santeon, Utrecht, The Netherlands.; ^3^Radboud University Medical Center, Nijmegen, The Netherlands.; ^4^Department of Value-Based Healthcare, St. Antonius Hospital, Nieuwegein, The Netherlands.

**Keywords:** Value-Based Healthcare, Performance Management Systems, Change Management, Organizational Structures

## Abstract

Value-based healthcare (VBHC) has emerged as a widely embraced strategy to address pressing healthcare challenges, including workforce shortages, rising healthcare costs, and inconsistent care quality. A scoping review by van Elten et al shows that despite their expected importance of integrating VBHC with performance management systems, very few articles provide concrete examples of this integration. Drawing on existing performance management literature, the authors explore possible reasons for why VBHC practitioners and researchers have largely overlooked this topic. This commentary critically engages with their review by examining their conceptual definitions, offering alternative explanations for the apparent lack of performance management in VBHC, and suggesting directions for future interdisciplinary research.

## Introduction

 Interest in management innovations aimed at addressing challenges related to unpredictable quality of care, insufficient coordination between healthcare domains, and increasing healthcare expenditure has grown rapidly in the last decade.^1^ One of the strategies that has been broadly embraced, is termed value-based healthcare (VBHC). In their article, van Elten and colleagues take a managerial lens towards this globally accepted strategy, and, in our view rightfully, state that the change towards VBHC has far-reaching organizational and managerial consequences.^2^ The authors put forward that the performance of VBHC initiatives *“ought to be strictly monitored”*^2^ (p. 2), which may happen with the use of performance management systems. In their scoping review, they aimed to summarize if and how VBHC is supported by performance management systems. Van Elten et al make an important contribution to the literature and propose an interesting viewpoint towards the integration and role of performance management systems in VBHC. However, we question a number of highlights and statements put forward by the authors, and want to further deepen the discussion on the integration of (performance) management systems in VBHC in this commentary, based on our combined expertise in the field of VBHC, and the articles that we have published on this topic.

## Comments on the Conceptualization of VBHC

 The authors state that, as hospitals are shifting their traditional siloed structures towards condition-based structures, it is important to learn how professionals manage this organizational shift. While we agree with the authors that a broader perspective is necessary when evaluating VBHC performance, their conceptualization of VBHC limits the clarity of their argument. Van Elten et al describe the potential benefits of VBHC and describe it as an innovation: *“an important innovation aimed at reforming healthcare practice and policy” *(p. 1).^2^ However, what is meant with such VBHC innovations, is not further specified. Previous research has already highlighted that many studies on VBHC fail to clearly define or articulate their perspective on the concept.^3,4^ Since Porter and Teisberg^5^ never fully conceptualized VBHC, the term has been interpreted and applied in varying ways throughout the literature. Some authors describe VBHC as equal to value in health, or the value equation,^6^ others consider VBHC a new overarching organizational structure,^7^ and others refer only to the goals of VBHC.^8^ This lack of clarity hinders the advancement of VBHC research.^3^ A more precise conceptual foundation is needed to enhance the field and ensure a more consistent understanding of how (performance) management systems should be integrated in VBHC practice. To maintain consistency in our own argument and enhance clarity regarding the VBHC concept in this current article, we adopt the conceptualization of VBHC as van Staalduinen^9^: *VBHC is a strategy aimed at maximizing patient value – defined as outcomes relative to patient costs – within cohesive collaborative structures organized around medical conditions covering all care disciplines involved. *

 In particular, in the study by van Elten et al, in addition to the conceptual clarity, we contend that two distinct performance measurement processes occur simultaneously but should be evaluated and implemented separately. Given that many hospitals adopt an incremental change approach to VBHC,^10^ VBHC elements have become a new and established way of working for some, but VBHC implementation remains an ongoing transformation process. In our view, these two processes require separate (performance) measurement approaches in both practice and research. To put it simply, we would distinguish between “measuring performance and progression in the transition to working according to VBHC principles” and “measuring performance with running the business according to VBHC principles.” However, the authors’ discussion does not clearly differentiate between these perspectives. Thereby, it remains unclear whether they are advocating for performance measurement in hospitals operating under VBHC principles or for evaluating the success of hospitals’ transition toward VBHC. We believe both is needed, but future research should deliberately address the perspective at hand. This distinction is crucial, as it affects how a VBHC study’s results are interpreted. When reading the results and interpretation of the results of the article of van Elten et al, we expect them to have studied the use of performance management systems for operating according to VBHC principles, but the distinction and choice would have been clearer with a more concrete explanation.

## Comments on the Findings

 Van Elten et al state in their results section that VBHC performance measurement appears to be limited in scope and is often confined to a single organization or department. They argue that VBHC is not as all-encompassing as originally suggested by Porter and Teisberg, a conclusion that aligns with findings from our previous scoping review.^3^

 The authors highlight three key elements of performance management—healthcare institutions’ key objectives, strategies, and reward systems—as emphasized in the performance management literature.^11^ They argue that these elements remain largely unaddressed in the VBHC literature. Their main argument for the absence of performance measurement systems in VBHC literature is that, although VBHC is intended to be implemented at the health system level, there is a lack of cross-organizational cooperation and no established framework for cross-organizational VBHC performance measurement. While we acknowledge that VBHC should ultimately guide a health system’s approach, we must challenge this argument, or at least, invite discussion on this matter. The authors state that “there is no one-size-fits-all approach to VBHC implementation,” yet they only consider “full-blown application of VBHC” suitable for performance measurement. We argue that small-scale VBHC initiatives offer valuable insights into effective performance management systems. Since VBHC is implemented incrementally,^10^ learning from small-scale performance management initiatives is crucial for its broader application. Additionally, while the authors mainly consider tensions between VBHC and performance measurement for medical specialists and administrators, they overlook VBHC’s growing role in the collaborative work of healthcare professionals. In condition-based units, professionals regularly assess their performance, using value-improvement cycles to refine their care delivery. Within Santeon, a collaborative network of seven Dutch hospitals, condition-based units have been established across the hospitals to systematically measure and improve patient outcomes. This is achieved by following a structured cycle of standardized data collection via scorecards, analyzing variations, and identifying and implementing targeted improvements. These VBHC initiatives not only enhance patient care but also reduce hospital costs by improving efficiency.^12,13^

 Building on the previous, we propose additional explanations for why performance measurement systems were not identified in the literature search by van Elten et al.

 First, while VBHC has been around for 15 years at the time of this review, it is still very much evolving. Despite the broad consensus that focusing on outcomes is the right approach, the journey is, thus, far from complete. Therefore, a reader must recognize the context of the healthcare system in which van Elten et al argue that performance management systems are crucial. This healthcare system has only recently begun measuring patient outcomes and costs to drive improvement initiatives focused on specific patient groups. In this current stage of VBHC development, hospitals simply do not yet have the correct and complete data to steer on performance. Key data such as patient reported outcome measures at the patient group level are not available to structurally measure performance. Also, we have yet to clearly define which key performance indicators are needed at different organizational levels. Dashboards for individual patient groups vary significantly, so some level of aggregation is necessary to align them with organizational goals. Thereby, there remains uncertainty about the best way to achieve this and a lot of work needs to be done in this area. Therefore, expecting performance management systems for VBHC to be fully implemented in hospitals, on a health system level, and for the full value chain, as suggested by van Elten et al, is, in our view, overly optimistic and premature.

 Second, starting in 2006, VBHC was an entirely new field for both research and practice, and as a result, relatively little was published on the topic in its early years. The research question as put forward by van Elten et al is: to what extent is VBHC supported by performance management systems in current practice? We argue that in this current practice, there was—and still is—more happening than what is reflected in the literature. Certain aspects of VBHC, such as measuring patient outcomes, healthcare professionals are already capable of defining some local objectives and end goals on an annual basis.^14^ These objectives, tailored to specific patient groups, provide a foundation for exploring meaningful performance measurement. Identifying these performance measurement systems, however, requires more than a surface-level examination of VBHC implementation methods described in the literature. This leads us to conclude that the timing of this study gives the impression that performance management is not in place in practice, overlooking existing initiatives in practice.

 Finally, redesigning a hospital organization according to VBHC principles is a highly complex process that affects the entire organization. Simply stating that *“performance measurement systems must be built to safeguard the progress of VBHC initiatives”*^2^ (p. 8) downplays the challenges involved. Measuring performance within a VBHC system raises a wide range of critical questions, particularly regarding the ability to effectively steer and manage outcomes. Given that both patient outcomes and process outcomes are central to VBHC, a key issue is determining who is responsible for these outcomes and who can be held accountable for achieving them. Furthermore, as hospitals transition to a VBHC structure, most organizations shift from a traditional, vertically organized system based on medical specialties to a more horizontally structured approach, where care delivery is increasingly organized around patient groups. This transformation often results in a matrix organization, in which teams from different disciplines collaborate within condition-based units.^15^ As a result, performance measurement and accountability shift from individual departments to multidisciplinary teams, making it essential to develop new steering mechanisms. Rather than focusing solely on departmental key performance indicators, organizations must establish ways to measure, compare, and improve outcomes at the team level. This shift requires clear governance, alignment with organizational goals, and effective data aggregation to ensure that performance measurement supports continuous improvement across patient pathways.

## Call for Action: Interdisciplinary Research on VBHC

 After reading the article by van Elten et al, it becomes clear there is a need to explore the integration of existing (traditional) hospital systems with the VBHC strategy. Recognizing the complexity of implementing VBHC in practice, we are eager to learn more about effectively integrating VBHC with performance management systems. We would have expected van Elten et al not only to highlight the necessity of this integration but also, given their expertise in the field, to provide concrete guidance on how to move forward. This leads us to end with a call to action, to collaborate and combine fields of expertise to improve VBHC adoption with the knowledge from the performance management literature. As changes due to VBHC impact a wide range of organizational aspects of the health system, we consider the implementation and effectiveness of VBHC to be enhanced through interdisciplinary collaboration between healthcare and management science.

## Concluding Remarks

 As hospitals worldwide scale up and advance in their implementation of VBHC, the first structural effects are becoming evident. Leading hospitals are now finding out that VBHC not only needs to be aligned with medical practice but also with existing organizational procedures and hospital operations. This growing recognition underscores the importance of exploring VBHC models’ integration with hospital systems, such as performance measurement systems, to enhance efficiency and patient outcomes.

## Ethical issues

 Ethical approval was deemed unnecessary, as this commentary reviews a previously published article and does not involve the use of newly collected data.

## Conflicts of interest

 Authors declare that they have no conflicts of interest.
